# Comparison between as-needed versus regular treatment for allergic rhinitis: Protocol for a systematic review 

**DOI:** 10.5414/ALX02654E

**Published:** 2026-09-25

**Authors:** Catarina Ferreira, Vítor Henrique Duarte, Nicky Akbarian, Antonio Bognanni, Sara Gil-Mata, Ana Margarida Pereira, Manuel Marques-Cruz, Torsten Zuberbier, Holger J. Schünemann, João A. Fonseca, Jean Bousquet, Rafael José Vieira, Bernardo Sousa-Pinto

**Affiliations:** 1 MEDCIDS – Department of Community Medicine, Information and Health Decision Sciences,; 2 CINTESIS@RISE – Health Research Network,; 3 Unit of Anatomy, Department of Biomedicine, Faculty of Medicine, University of Porto, Porto, Portugal,; 4 Faculty of Medicine, University of Ottawa, Ottawa, ON, Canada,; 5 Clinical Epidemiology and Research Center (CERC), IRCCS Humanitas Research Hospital & Department of Biomedical Sciences, Humanitas University, Milan, Italy,; 6 Institute of Allergology, Charité – Universitätsmedizin Berlin, Corporate Member of Freie Universität Berlin and Humboldt-Universität zu Berlin,; 7 Fraunhofer Institute for Translational Medicine and Pharmacology ITMP, Immunology and Allergology, Berlin, Germany, and; 8 ARIA, Montpellier, France

**Keywords:** allergic rhinitis, antihistamines, intranasal corticosteroids, as-needed treatment

## Abstract

Background: Allergic rhinitis (AR) impairs quality of life. While most trials assess regular regimen medication use, mobile-Health (mHealth) data suggest that most patients use treatments on an as-needed basis. Objectives: Protocol for a systematic review comparing as-needed versus regular AR treatment regimens regarding efficacy and safety. Materials and methods: We will conduct a systematic review and network meta-analysis (NMA) of randomized controlled trials involving patients of any age with perennial or seasonal AR. Oral and intranasal drugs will be compared in an as-needed regimen versus a regular regimen or placebo. Primary outcomes will be the Total Nasal Symptom Score, the Total Ocular Symptom Score, and the Rhinoconjunctivitis Quality-of-Life Questionnaire. Conclusion: This review will evaluate the efficacy and safety of two different strategies for AR management. The findings of this review will inform the Allergic Rhinitis and its Impact on Asthma (ARIA) 2024-2025 guidelines.

## Introduction 

Allergic rhinitis (AR) is an inflammatory disorder of the nasal mucosa mediated by Immunoglobulin E (IgE) after exposure to antigens [1]. It is characterized by nasal symptoms (including rhinorrhea, nasal congestion, nasal pruritus, and/or sneezing [2]), ocular symptoms (eye itching, watery eyes, red eyes) [3], and other symptoms. AR is a very prevalent condition [4] that affects all age groups and has an important impact on quality of life and productivity [5, 6, 7, 8, 9]. 

There are several medication classes that can be used in the management of AR [10], including oral antihistamines (OAH), intranasal corticosteroids (INCS), intranasal antihistamines (INAH), and fixed combinations of INAH+INCS. Most trials on such medications have assessed their use on a regular regimen; that is, with patients taking medication on a daily basis. However, studies based on mobile-Health (mHealth) direct patient data suggest that most patients use their medications on an as-needed basis [11]. That is, patients tend to take medication for AR when not feeling sufficiently well controlled [12]. The same has been observed even among allergists with AR [13]. 

There is limited evidence on the impact of the as-needed use of medication on the control of AR. Previous systematic reviews on this topic [14, 15, 16] have some limitations, considering that they have only included studies (i) directly comparing these two treatment regimens, and (ii) evaluating intranasal drugs. In this regard, there is a need for multiple comparison analyses that compare the as-needed and the regular treatment regimens of intranasal and oral drugs. 

This article describes the protocol of a systematic review with network meta-analysis (NMA) that will aim to compare the efficacy of the as-needed versus regular treatment regimens in patients with AR. In particular, we want to evaluate whether an as-needed regimen is associated with differences in terms of nasal symptoms, ocular symptoms, and rhinoconjunctivitis-related quality of life compared to regular treatment. Additionally, we will compare the safety of both strategies. This systematic review will inform the Allergic Rhinitis and its Impact on Asthma (ARIA) 2024 – 2025 guidelines. 

## Materials and methods 

This systematic review will follow the Preferred Reporting Items for Systematic Reviews and Meta-Analyses (PRISMA) extension for NMA (PRISMA-NMA) [17]. Its protocol has been prepared following the PRISMA for protocols (PRISMA-P) and registered in PROSPERO (CRD420251128995). 

### Eligibility criteria 

We will include randomized clinical trials (RCTs) with a parallel design involving patients of any age with perennial or seasonal AR. The interventions that will be evaluated include (i) intranasal medications (corticosteroids, antihistamines, or their fixed combinations), (ii) oral medications (antihistamines or antileukotriene receptor antagonists) for AR, or (iii) placebo. We will consider the above cited active treatments used in an as-needed or regular regimen. We will consider an as-needed regimen when patients opt for using medication depending on their symptoms, and we consider a regular regimen when the frequency of treatment administration is fixed and occurs daily regardless of symptom severity on that time. The use of rescue medication in addition to the regular treatment for which participants have been allocated will not be considered as an as-needed regimen and will not be analyzed. 

In light of the outcomes prioritized by the ARIA 2024 – 2025 guideline panel, we will include studies reporting results on at least one of the following patient-reported outcome measures (PROM), which correspond to our main outcomes: Total Nasal Symptom Score (TNSS), Total Ocular Symptom Score (TOSS), or Rhinoconjuctivitis Quality-of-Life Questionnaire (RQLQ). We define the TNSS as any score computed based on the sum of four individual patient-reported scores for nasal symptoms (nasal congestion, rhinorrhea, nasal itching, and sneezing) and the TOSS as any score computed based on the sum of three individual patient-reported scores for ocular symptoms (itching/burning, tearing/watering, and redness). We will standardize different scales used by converting them into the same scale (e.g., we will convert TNSS scales of 0 – 400 to scales of 0 – 12) as described in previous systematic reviews by our team [18, 19]. Our secondary outcomes will be the frequency of (i) developing at least 1 adverse event (AE) or (ii) developing at least 1 serious AE, and (iii) treatment withdrawal due to an AE. 

To be included, RCTs will need to have a follow-up of at least 2 weeks for seasonal AR and 4 weeks for perennial AR, in line with the recommendations of the U.S. Food and Drug Administration [20]. RCTs on seasonal AR in which the intervention starts before the pollen season will be excluded. 

### Information sources and search strategy 

We will conduct a literature search to identify eligible RCTs. We will search the following electronic bibliographic databases: MEDLINE (via Ovid), Embase, Cochrane Central Register of Controlled Trials (CENTRAL), and Web of Science (WoS). No restrictions on language, publication date or publication status will be applied. To access unpublished data, we will complement our search in electronic bibliographic databases with searches on databases of clinical trials (clinicaltrials.gov, the GSK clinical study dataset, and the AstraZeneca Clinical Trials Website) and with references from other published systematic reviews. 

The search strategy (Supplemental Table 1) will combine controlled vocabulary and free-text terms for:

- Interventions – including intranasal antihistamines (e.g., azelastine, olopatadine, levocabastine, bilastine, cetirizine, loratadine, fexofenadine), intranasal corticosteroids (e.g., fluticasone propionate, mometasone, budesonide, beclomethasone, ciclesonide, triamcinolone), and fixed-dose combinations (e.g., azelastine-fluticasone).
- Disease – allergic rhinitis and rhinoconjunctivitis.
- Study design filters – validated filters for randomized controlled trials in humans.
- Dosing regimens – search terms related to as-needed use (“as needed”, “PRN”, “on demand”, “rescue”) and regular use (“maintenance”, “continuous”, “continued”).

### Study selection and data collection 

The study selection will be made independently by two researchers, initially by title and abstract screening and later by reading the full text. 

We will verify, for every included record, whether multiple publications resulting from the same study exist, to ensure that participant information is not duplicated. 

For each included record, two researchers will independently extract data on variables related to: (1) type of disease (seasonal vs. perennial AR), (2) inclusion and exclusion criteria of the patients, (3) period of data collection, (4) patients’ recruitment location, (5) treatment regimen (regular or as-needed) and respective medications, (6) duration of follow up, (7) number, age and sex distribution of randomized participants, (8) baseline values and change-from-baseline values in the assessed PROM (TNSS, TOSS, and RQLQ), and (9) safety data (number of patients developing at least 1 AE or 1 serious AE, and number of patients who discontinued the treatment because of an AE). We will also collect the data required to assess the studies’ risk of bias. Whenever results are only provided in a graphical form (rather than numerical data in text form), estimates will be obtained using the PlotDigitizer tool. 

Disagreements in study inclusion, data selection, or extraction will be resolved by consensus or, if necessary, by a third reviewer. 

### Risk of bias and certainty assessment 

Risk of bias in each primary study will be evaluated independently by two reviewers at the outcome level using the Cochrane risk-of-bias tool (2011 version) [21], following the methodology for the other systematic reviews for the ARIA 2024 – 2025 guidelines [22], with conflicts resolved by consensus or a third reviewer. 

Certainty of evidence will be judged at an outcome level using the Grading of Recommendations Assessment, Development, and Evaluation (GRADE) working group approach for NMA [23, 24]. Applying GRADE implies setting decision thresholds, that is, reference values that indicate whether an effect size is small, moderate, or large. For continuous outcomes, we will consider the standardized mean differences of 0.2, 0.5, and 0.8 as decision thresholds; These thresholds respectively distinguish “trivial or none” from “small” effects, “small” from “moderate” effects, and “moderate” from “large” effects [25]. Consequently, meta-analytical measures for continuous outcomes will be recalculated as standardized mean differences for the purpose of certainty of evidence assessment. For dichotomous outcomes, we will consider previously determined decision thresholds for AEs [26]. 

### Quantitative synthesis of results 

We will perform NMA for continuous (TNSS, TOSS, and RQLQ) and categorical (safety-related outcomes) outcomes. For continuous outcomes, we will perform random-effects NMA of mean differences in change-from-baseline values (to support interpretability of results, the NMA will be conducted with mean differences and the GRADE certainty of evidence assessment will be conducted based on standardized mean differences). Any missing information on spread measures (standard deviation, standard error, or variance) will be estimated using the algorithm suggested by Weir et al. [27], as previously described [28]. For categorical outcomes, we will perform random-effects NMA of risk ratios. 

We will perform an NMA in order to simultaneously compare multiple interventions even in the absence of studies that compare them directly [29]. An NMA can combine direct and indirect evidence and will make it possible to estimate efficacy and compare the safety of different interventions. 

In the context of our review, an NMA will permit the comparison between (i) interventions in an as-needed regimen, (ii) interventions in a regular regimen, and (iii) placebo. For instance, if some studies compare the as-needed regimen to a placebo and others compare the regular regimen to placebo as well, the NMAs will permit the (indirect) comparison between the two regimens. Such comparisons will be performed at a medication class level (i.e., INCS, INAH, INHA+INCS and OAH) and, if possible, at an individual medication level, with each node corresponding to a pair “treatment-regimen” (e.g., INCS on a regular basis and INCS on an as-needed basis will correspond to different nodes of the NMA). We will do sensitivity analysis according to AR type (i.e., seasonal or perennial) and population age (i.e., adults or children). For follow-up, considering U.S. Food and Drug Administration recommendations [30], we will prioritize data obtained at 2 weeks for seasonal AR and 4 weeks for perennial AR [22]. When such data are not available, we will use data from the end of the follow-up period [22]. 

The transitivity assumption (patients in different studies could have been randomized to any of the treatments to be compared, so the patients’ characteristics and study designs must be comparable) will be evaluated by evaluating the studies’ characteristics. The coherence assumption (the results from both direct and indirect comparisons must agree) will be evaluated based on the hypothesis test that compares direct and indirect estimates: at the network level, we will evaluate the result of the incoherence test (with p-values < 0.05 being indicative of significant incoherence); at the comparison level, we will evaluate whether direct and indirect estimates are coherent by adopting a Separating Indirect from Direct Evidence (SIDE) approach [31]. Heterogeneity (the degree of variation between studies addressing the same comparison, beyond what is expected by chance) will be evaluated using the I^2^ statistic – heterogeneity will be considered substantial if the I^2^ statistic is higher than 50%. 

The analyses will be made using the software R. 

### Ethics and dissemination 

Ethical approval will not be required. Findings will be published in a peer-reviewed journal. Data will be available from the corresponding author upon reasonable request. 

## Discussion 

This systematic review will compare the efficacy and safety of two treatment strategies used in AR: as-needed vs. regular medication use. While regular medication use is the strategy most commonly evaluated in randomized controlled trials, studies based on direct patient data suggest that most patients use their treatments on as-needed basis [11]. 

There are some limitations to our systematic review. AR can present with different levels of severity (from mild to severe forms), and there may be differences in efficacy of the “as-needed” vs. regular regimens depending on the severity of the disease. Most likely, we will not be able to evaluate these differences since it is unlikely that primary studies provide data stratified by baseline severity. Moreover, in most RCTs, patients with severe forms of the disease tend to be over-represented. 

Our systematic review can be particularly relevant in guiding future guidelines. If no differences are demonstrated, future recommendations may be more aligned with the patients’ behavioral patterns regarding drug regimens. In addition, recommending an as-needed strategy can also have economic and planetary health benefits – lower medication use can result in decreased costs both for the patient and society and in decreased use of resources (leading to less harmful greenhouse gas emissions). 

## Authors’ contributions 

Catarina Ferreira, Vítor Henrique Duarte and Nicky Akbharian participated in the methodological design of the protocol and in the writing of the first draft of the manuscript. 

Antonio Bognanni, Sara Gil-Mata, Ana Margarida Pereira, Manuel Marques-Cruz, Torsten Zuberbier, Holger J. Schünemann, João A. Fonseca, Jean Bousquet, Rafael José Vieira, Bernardo Sousa-Pinto participated in the methodological design of the protocol and in the critical revision of the manuscript. 

## Funding 

This research will not receive any funding. 

## Conflict of interest 

J.A. Fonseca reports grants from AstraZeneca and Mundipharma; and personal fees from AstraZeneca, Mundipharma, Sanofi, GSK, and Teva outside the submitted work. T. Zuberbier reports grants from Novartis and Henkel; and personal fees from Bayer Health Care, FAES, Novartis, Henkel, AstraZeneca, AbbVie, ALK-Abelló, Almirall, Astellas, Bencard, Berlin Chemie, HAL, Leti, Meda, Menarini, Merck, MSD, Pfizer, Sanofi, Stallergenes, Takeda, Teva, UCB, Kryolan, and L’Oréal outside the submitted work. J. Bousquet reports personal fees from Chiesi, Cipla, Hikma, Menarini, Mundipharma, Mylan, Novartis, Purina, Sanofi-Aventis, Takeda, Teva, and Uriach; and other from Kyomed-Innov outside the submitted work. The rest of the authors declare that they have no relevant conflicts of interest. 

## Supplemental material

Supplemental material Supplementary tables

## References

[1] Bousquet J Khaltaev N Cruz AA Denburg J Fokkens WJ Togias A Zuberbier T Baena-Cagnani CE Canonica GW van Weel C Agache I Aït-Khaled N Bachert C Blaiss MS Bonini S Boulet LP Bousquet PJ Camargos P Carlsen KH Chen Y Allergic Rhinitis and its Impact on Asthma (ARIA) 2008 update (in collaboration with the World Health Organization, GA(2)LEN and AllerGen). Allergy. 2008; 63: 8–160. 10.1111/j.1398-9995.2007.01620.x 18331513

[2] Siddiqui ZA Walker A Pirwani MM Tahiri M Syed I Allergic rhinitis: diagnosis and management. Br J Hosp Med (Lond). 2022; 83: 1–9. 10.12968/hmed.2021.0570 35243888

[3] Lee JE Kim KR Rha KS Dhong HJ Roh HJ Rhee CS Kim KS Park DJ Kim SW Kim YD Lim SC Ahn BH Kang JM Lee JH Kim CH Kim SH Kim HJ Lee KH Cho KS Jung YH Prevalence of ocular symptoms in patients with allergic rhinitis: Korean multicenter study. Am J Rhinol Allergy. 2013; 27: e135–e139. 10.2500/ajra.2013.27.3937 24119594

[4] Brożek JL Bousquet J Agache I Agarwal A Bachert C Bosnic-Anticevich S Brignardello-Petersen R Canonica GW Casale T Chavannes NH Correia de Sousa J Cruz AA Cuello-Garcia CA Demoly P Dykewicz M Etxeandia-Ikobaltzeta I Florez ID Fokkens W Fonseca J Hellings PW Allergic Rhinitis and its Impact on Asthma (ARIA) guidelines-2016 revision. J Allergy Clin Immunol. 2017; 140: 950–958. 10.1016/j.jaci.2017.03.050 28602936

[5] Vieira RJ Pham-Thi N Anto JM Czarlewski W Sá-Sousa A Amaral R Bedbrook A Bosnic-Anticevich S Brussino L Canonica GW Cecchi L Cruz AA Fokkens WJ Gemicioglu B Haahtela T Ivancevich JC Klimek L Kuna P Kvedariene V Larenas-Linnemann D Academic Productivity of Young People With Allergic Rhinitis: A MASK-air Study. J Allergy Clin Immunol Pract. 2022; 10: 3008–3017. 10.1016/j.jaip.2022.08.015 35998876

[6] Vieira RJ Azevedo LF Pereira AM Nogueira-Leite D Rocha Gonçalves FN Larenas-Linnemann DE Cruz AA Gemicioglu B Samolinski B de Las Vecillas L Giovannini M Cunha MJ Rodrigues J Kvedariene V Klimek L Pfaar O Zuberbier T Fonseca JA Bousquet J Sousa-Pinto B Impact of Allergic Rhinitis Control on Work Productivity and Costs: A Real-World Data MASK-air Study. J Allergy Clin Immunol Pract. 2024; 12: 3107–3115. 10.1016/j.jaip.2024.07.026 39111363

[7] Vieira RJ Pereira AM Kupczyk M Regateiro FS Larenas-Linnemann DE Toppila-Salmi S Iinuma T Kuna P Cruz AA Brussino L Gemicioglu B Samolinski B Taborda-Barata L Ventura MT Kvedariene V Klimek L Pfaar O Zuberbier T Azevedo LF Fonseca JA Impact of allergic symptoms on work productivity in allergic rhinitis: A MASK-air direct patient data study. Allergol Int. 2025; 74: 445–452. 10.1016/j.alit.2024.12.007 40175264

[8] Vieira RJ Leemann L Briggs A Pereira AM Savouré M Kuna P Morais-Almeida M Bewick M Azevedo LF Louis R Klimek L Bahbah F Samolinski B Anto JM Zuberbier T Fonseca JA Bousquet J Sousa-Pinto B Czarlewski W Bedbrook A Poor Rhinitis and Asthma Control Is Associated With Decreased Health-Related Quality of Life and Utilities: A MASK-air Study. J Allergy Clin Immunol Pract. 2024; 12: 1530–1538. 10.1016/j.jaip.2024.03.036 38561141

[9] Vieira RJ Leemann L Schünemann HJ Azevedo LF Fonseca JA Bousquet J Sousa-Pinto B Impact of Uncontrolled Symptoms on the Health-Related Quality of Life (EQ-5D-5L) of Patients With Allergic Rhinitis: A MASK-air Study. Clin Exp Allergy. 2024; 54: 634–637. 10.1111/cea.14516 39031606

[10] Bjermer L Westman M Holmström M Wickman MC The complex pathophysiology of allergic rhinitis: scientific rationale for the development of an alternative treatment option. Allergy Asthma Clin Immunol. 2019; 15: 24. 10.1186/s13223-018-0314-1 PMC6469109 31015846

[11] Sousa-Pinto B Fonseca JA Bousquet J Contribution of MASK-air(R) as a mHealth tool for digitally-enabled person-centred care in rhinitis and asthma. J Investig Allergol Clin Immunol. 2024; online ahead of print. 10.18176/jiaci.0994 38745438

[12] Sousa-Pinto B Sá-Sousa A Vieira RJ Amaral R Klimek L Czarlewski W Antó JM Pfaar O Bedbrook A Kvedariene V Ventura MT Ansotegui IJ Bergmann KC Brussino L Canonica GW Cardona V Carreiro-Martins P Casale T Cecchi L Chivato T Behavioural patterns in allergic rhinitis medication in Europe: A study using MASK-air real-world data. Allergy. 2022; 77: 2699–2711. 10.1111/all.15275 35258105

[13] Bousquet J Murray R Price D Somekh D Münter L Phillips J Czarlewski W The allergic allergist behaves like a patient. Ann Allergy Asthma Immunol. 2018; 121: 741–742. 10.1016/j.anai.2018.07.034 30056150

[14] Phinyo P Wongsa C Sompornrattanaphan M Thongngarm T As-needed versus regular intranasal corticosteroid for allergic rhinitis: A systematic review and meta-analysis. Asian Pac J Allergy Immunol. 2022; 40: 195–204. 10.12932/AP-091121-1269 35278059

[15] Hoang MP Chitsuthipakorn W Seresirikachorn K Snidvongs K As-needed intranasal corticosteroid spray for allergic rhinitis: a systematic review and meta-analysis. Rhinology. 2022; 60: 242–251. 10.4193/Rhin21.355 35379997

[16] Laekeman G Simoens S Buffels J Gillard M Robillard T Benedetti MS Watelet JB Liekendael G Ghys L Church M Continuous versus on-demand pharmacotherapy of allergic rhinitis: evidence and practice. Respir Med. 2010; 104: 615–625. 10.1016/j.rmed.2010.01.006 20163944

[17] Hutton B Salanti G Caldwell DM Chaimani A Schmid CH Cameron C Ioannidis JP Straus S Thorlund K Jansen JP Mulrow C Catalá-López F Gøtzsche PC Dickersin K Boutron I Altman DG Moher D The PRISMA extension statement for reporting of systematic reviews incorporating network meta-analyses of health care interventions: checklist and explanations. Ann Intern Med. 2015; 162: 777–784. 10.7326/M14-2385 26030634

[18] Sousa-Pinto B Vieira RJ Brozek J Cardoso-Fernandes A Lourenço-Silva N Ferreira-da-Silva R Ferreira A Gil-Mata S Bedbrook A Klimek L Fonseca JA Zuberbier T Schünemann HJ Bousquet J Intranasal antihistamines and corticosteroids in allergic rhinitis: A systematic review and meta-analysis. J Allergy Clin Immunol. 2024; 154: 340–354. 10.1016/j.jaci.2024.04.016 38685482

[19] Sousa-Pinto B Vieira RJ Bognanni A Gil-Mata S Ferreira-da-Silva R Ferreira A Cardoso-Fernandes A Ferreira-Cardoso H Marques-Cruz M Duarte VH Castro-Teles J Campos-Lopes M Teixeira-Ferreira A Lourenço-Silva N Chérrez-Ojeda I Bedbrook A Klimek L Nuñez JJY Zuberbier T Fonseca JA Efficacy and safety of intranasal medications for allergic rhinitis: Network meta-analysis. Allergy. 2025; 80: 94–105. 10.1111/all.16384 39548801

[20] Food and Drug Administration. Allergic Rhinitis: Developing Drug Products for Treatment – Guidance for Industry. 2018.

[21] Higgins JP Altman DG Gøtzsche PC Jüni P Moher D Oxman AD Savovic J Schulz KF Weeks L Sterne JA The Cochrane Collaboration’s tool for assessing risk of bias in randomised trials. BMJ. 2011; 343 (oct18 2): d5928 10.1136/bmj.d5928 PMC3196245 22008217

[22] Vieira RJ Torres MI Bognanni A Gil-Mata S Ferreira-da-Silva R Lourenço-Silva N Cardoso-Fernandes A Ferreira A Ferreira-Cardoso H Teles J Campos-Lopes M Fonseca JA Yepes-Nuñez JJ Klimek L Zuberbier T Schünemann H Bousquet J Sousa-Pinto B Protocol for the systematic reviews on the desirable and undesirable effects of pharmacological treatments of allergic rhinitis informing the ARIA 2024 guidelines. Allergol Select. 2024; 8: 270–277. 10.5414/ALX02515E PMC11270335 39055747

[23] Brignardello-Petersen R Florez ID Izcovich A Santesso N Hazlewood G Alhazanni W Yepes-Nuñez JJ Tomlinson G Schünemann HJ Guyatt GH GRADE approach to drawing conclusions from a network meta-analysis using a minimally contextualised framework. BMJ. 2020; 371: m3900. 10.1136/bmj.m3900 33177059

[24] Brignardello-Petersen R Izcovich A Rochwerg B Florez ID Hazlewood G Alhazanni W Yepes-Nuñez J Santesso N Guyatt GH Schünemann HJ GRADE approach to drawing conclusions from a network meta-analysis using a partially contextualised framework. BMJ. 2020; 371: m3907. 10.1136/bmj.m3907 33172877

[25] Cohen J Statistical power analysis for the behavioral sciences. New York: Routledge; 2013.

[26] Sousa-Pinto B Bognanni A Gil-Mata S Vieira RJ Xie F Bonovas S Piovani D Morgano GP Ferreira-da-Silva R Ferreira A Cardoso-Fernandes A Lourenço-Silva N Piggott T Wiercioch W Neumann I Bedbrook A Yepes-Nuñez JJ Klimek L Zuberbier T Fonseca JA Empirical estimation of disutilities and decision thresholds for composite endpoints. J Clin Epidemiol. 2025; 179: 111638. 10.1016/j.jclinepi.2024.111638 39662643

[27] Weir CJ Butcher I Assi V Lewis SC Murray GD Langhorne P Brady MC Dealing with missing standard deviation and mean values in meta-analysis of continuous outcomes: a systematic review. BMC Med Res Methodol. 2018; 18: 25. 10.1186/s12874-018-0483-0 PMC5842611 29514597

[28] Sousa-Pinto B Vieira RJ Brozek J Cardoso-Fernandes A Lourenço-Silva N Ferreira-da-Silva R Ferreira A Gil-Mata S Bedbrook A Klimek L Fonseca JA Zuberbier T Schünemann HJ Bousquet J Intranasal antihistamines and corticosteroids in the treatment of allergic rhinitis: a systematic review and meta-analysis protocol. BMJ Open. 2023; 13: e076614. 10.1136/bmjopen-2023-076614 PMC10626820 37918935

[29] Watt J Del Giovane C Network Meta-Analysis. Methods Mol Biol. 2022; 2345: 187–201. 10.1007/978-1-0716-1566-9_12 34550592

[30] Administration FaD. Allergic rhinitis: developing drug products for treatment – guidance for industry. 2018.

[31] Dias S Welton NJ Caldwell DM Ades AE Checking consistency in mixed treatment comparison meta-analysis. Stat Med. 2010; 29: 932–944. 10.1002/sim.3767 20213715

