# Supplementary material for: Comparison between as-needed versus regular treatment for allergic rhinitis: Protocol for a systematic review
Source: Allergol Select. 2026 Sep 25;10:296–302. doi: 10.5414/ALX02654E (PMC13647244; doi:10.5414/ALX02654E)
Supplement: Supplementary file 1 [file allergologieselect-10-296-S01.pdf]

Supplementary table

Supplementary table 1 – queries used for the different databases

Medline Ovid

1. azelastin$.mp.
2. azelastine-fluticasone.mp.
3. azeflu.mp.
4. MP-azeflu.mp.
5. (azelastine adj2 fluticasone).mp.
6. dymista.mp.
7. azecort.mp.
8. olopatadin$.mp.
9. olopatadine-mometasone.mp.
10. (olopatadine adj2 mometasone).mp.
11. gsp301.mp.
12. ryaltris.mp.
13. levocabastin$.mp.
14. lexicomp.mp.
15. ciclesonid$.mp.
16. Alvesco.mp.
17. fluticason$.mp.
18. GW685698X.mp.
19. mometason$.mp.
20. beclometason$.mp.
21. budesonid$.mp.
22. triamcinolon$.mp.
23. azelastine.nm.
24. levocabastine.nm.
25. ciclesonide.nm.
26. acrivastine$.mp.
27. alimemazine$.mp.
28. antazoline$.mp.
29. astemizole$.mp.
30. bilastine$.mp.
31. brompheniramine$.mp.
32. carbinoxamine$.mp.
33. cetirizine$.mp.
34. chlorpheniramine$.mp.
35. clemastine$.mp.
36. cetrizine$.mp.
37. cyproheptadine$.mp.
38. desloratadine$.mp.
39. dexbrompheniramine$.mp.
40. dexchlorpheniramine$.mp.
41. dimenhydrinate$.mp.
42. dimethindene$.mp.
43. diphenhydramine$.mp.
44. doxylamine$.mp.
45. ebastine$.mp.
46. epinastine$.mp.
47. fexofenadine$.mp.
48. hydroxyzine$.mp.

49. loratadine$.mp.
50. meclizine$.mp.
51. mequitazine$.mp.
52. methapyrilene$.mp.
53. mizolastine$.mp.
54. oxatomide$.mp.
55. oxomemazine$.mp.
56. pheniramine$.mp.
57. promethazine$.mp.
58. pyrilamine$.mp.
59. quifenadine$.mp.
60. rupatadine$.mp.
61. terfenadine$.mp.
62. trimeprazine$.mp.
63. tripelennamine$.mp.
64. triprolidine$.mp.
65. exp Olopatadine Hydrochloride/
66. exp Fluticasone/
67. exp Mometasone Furoate/
68. exp Beclomethasone/
69. exp Budesonide/
70. exp Triamcinolone/
71. exp Astemizole/
72. exp Cetirizine/
73. exp Chlorpheniramine/
74. exp Cyproheptadine/
75. exp Clemastine/
76. exp Hydroxyzine/
77. exp Loratadine/
78. exp Ketotifen/
79. exp Promethazine/
80. exp Terfenadine/
81. exp Antazoline/
82. exp Methapyrilene/
83. exp Pyrilamine/
84. exp Tripelennamine/
85. exp Dimenhydrinate/
86. exp Diphenhydramine/
87. exp Doxylamine/
88. exp Brompheniramine/
89. exp Dimethindene/
90. exp Pheniramine/
91. exp Triprolidine/
92. exp Meclizine/ or exp Trimeprazine/
93. exp Rhinitis/
94. (rhinit$ or rhinoconjunctivit$).mp.
95. exp Allergic Rhinitis/
96. (hayfever or "hay fever" or pollenosis or pollinosis or SAR).mp.
97. or/1-92
98. or/93-96
99. 97 and 98
100. exp randomized controlled trial/
101. controlled clinical trial.pt.
102. randomized.ab.

103. placebo.ab.
104. clinical trials as topic/
105. randomly.ab.
106. trial.ti.
107. or/100-106
108. exp animals/ not humans/
109. 107 not 108
110. 99 and 109
111. prn.mp.
112. “pro re nata”.mp.
113. “on demand”.ab.
114. On-demand.ab.
115. (as adj1 needed).ab.
116. (as adj1 required).ab.
117. Rescue.ab.
118. (maintenance or continuous or continued).ab.
119. or/111-118
120. 110 and 119

OVID - Embase

#	Searches	Results
1	(azelastin* or 'azelastine-fluticasone' or azeflu or 'MP-azeflu' or (azelastine adj2 fluticasone) or dymista or azecort or olopatadin* or 'olopatadine-mometasone' or (olopatadine adj2 mometasone) or 'gsp301 or ryaltris' or levocabastin* or lexicomp or ciclesonid* or Alvesco or fluticason* or GW685698X or mometason* or beclometason* or budesonid* or triamcinolon* or antazoline* or methapyrilene* or pyrilamine* or tripelennamine* or clemastine* or dimenhydrinate* or diphenhydramine* or doxylamine* or brompheniramine* or chlorpheniramine* or dimethindene* or pheniramine* or triprolidine* or promethazine* or cetrizine* or meclizine* or hydroxyzine* or astemizole* or cyproheptadine* or loratadine* or terfenadine* or acrivastine* or fexofenadine* or dexbrompheniramine* or carbinoxamine* or bilastine* or cetirizine* or desloratadine* or dexchlorpheniramine* or dimetindene* or diphenhydramine* or ebastine* or epinastine* or levocabastine* or rupatadine* or mizolastine* or mequitazine* or alimemazine* or trimeprazine* or oxatomide* or oxomemazine* or quifenadine*).ti,ab,tn.	68454
2	(rhinit* or rhinoconjunctivit* or hayfever or "hay fever" or pollenosis or pollinosis).ti,ab,tn.	59955
3	1 and 2	5083
4	exp randomised controlled trial/	1094109
5	exp controlled clinical trial/	1284227
6	random*.ti,ab.	2469041
7	exp randomization/	101540
8	exp intermethod comparison/	315575

9	placebo.ti,ab.	461139
10	(compare or compared or comparison).ti.	686177
11	((evaluated or evaluate or evaluating or assessed or assess) and (compare or compared or comparing or comparison)).ab.	3299802
12	(open adj1 label).ti,ab.	185681
13	((double or single or doubly or singly) adj1 (blind or blinded or blindly)).ti,ab.	365985
14	exp double blind procedure/	306528
15	'parallel group$'.ti,ab.	52575
16	(crossover or 'cross over').ti,ab.	155397
17	((assign* or match or matched or allocation) adj5 (alternate or group$ or intervention$ or patient$ or subject$ or participant$)).ti,ab.	508939
18	(assigned or allocated).ti,ab.	602056
19	(controlled adj7 (study or design or trial)).ti,ab.	628074
20	(volunteer or volunteers).ti,ab.	321266
21	exp human experiment/	723764
22	trial.ti.	554399
23	or/4-22	7442881
24	(((case adj1 control*) and random*) not 'randomi?ed controlled').ti,ab.	24473
25	('systematic review' not (trial or study)).ti.	334615
26	(nonrandom* not random*).ti,ab.	21210
27	"Random field*".ti,ab.	3201
28	('random cluster' adj3 sampl*).ti,ab.	1783
29	(review.ab. and review.ti.) not trial.ti.	565589
30	"we searched".ab. and (review.ti. or review.ab.)	59438
31	"update review".ab.	154
32	(databases adj4 searched).ab.	77884
33	(rat or rats or mouse or mice or swine or porcine or murine or sheep or lambs or pigs or piglets or rabbit or rabbits or cat or cats or dog or dogs	1312384

	or cattle or bovine or monkey or monkeys or trout or marmoset$).ti. and exp animal experiment/	
34	exp animal experiment/ not (exp human experiment/ or exp human/)	2767733
35	or/24-34	3592910
36	23 not 35	6737738
37	3 and 36	2976
38	(prn or "pro re nata" or "on demand" or "on-demand" or "as-needed" or "as needed" or rescue or "as required" or "as-required" or maintenance or continuous or continued).ti,ab.	4582986
39	37 and 38	420

Web of Science Core Collection

Entitlements	Search Query
- WOS.IC: 1993 to 2025<br>- WOS.CCR: 1985 to 2025<br>- WOS.SCI: 1900 to 2025<br>- WOS.AHCI: 1975 to 2025<br>- WOS.ESCI: 2020 to 2025<br>- WOS.ISTP: 1990 to 2025<br>- WOS.SSCI: 1956 to 2025<br>- WOS.ISSHP: 1990 to 2025	TS=(azelastin* OR azelastine-fluticasone OR azeflu OR MP-azeflu OR (azelastine NEAR/2 fluticasone) OR dymista OR azecort OR olopatadin* OR olopatadine-mometasone OR (olopatadine NEAR/2 mometasone) OR gsp301 OR ryaltris OR levocabastin* OR lexicomp OR ciclesonid* OR Alvesco OR fluticason* OR GW685698X OR mometason* OR beclometason* OR budesonid* OR triamcinolon* OR antazoline* or methapyrilene* OR pyrilamine* OR tripelennamine* OR clemastine* OR dimenhydrinate* OR diphenhydramine* OR doxylamine* OR brompheniramine* OR chlorpheniramine* OR dimethindene* OR pheniramine* OR triprolidine* OR promethazine* OR cetrizine* OR meclizine* OR hydroxyzine* OR astemizole* OR cyproheptadine* OR loratadine* OR terfenadine* OR acrivastine* OR fexofenadine* OR dexbrompheniramine* OR carbinoxamine* OR bilastine* OR cetirizine* OR desloratadine* OR dexchlorpheniramine* OR dimetindene* OR diphenhydramine* OR ebastine* OR epinastine* OR levocabastine* OR rupatadine* OR mizolastine* OR mequitazine* OR alimemazine* OR trimeprazine* OR oxatomide* OR oxomemazine* OR quifenadine*)
- WOS.IC: 1993 to 2025<br>- WOS.CCR: 1985 to 2025<br>- WOS.SCI: 1900 to 2025<br>- WOS.AHCI: 1975 to 2025<br>- WOS.ESCI: 2020 to 2025<br>- WOS.ISTP: 1990	TS=(rhinit* or rhinoconjunctivit*) OR TS=(hayfever or "hay fever" or pollenosis or pollinosis or SAR)

to 2025<br>- WOS.SSCI: 1956 to 2025<br>- WOS.ISSHP: 1990 to 2025	
- WOS.IC: 1993 to 2025<br>- WOS.CCR: 1985 to 2025<br>- WOS.SCI: 1900 to 2025<br>- WOS.AHCI: 1975 to 2025<br>- WOS.ESCI: 2020 to 2025<br>- WOS.ISTP: 1990 to 2025<br>- WOS.SSCI: 1956 to 2025<br>- WOS.ISSHP: 1990 to 2025	TS=(randomised OR randomised OR randomisation OR randomisation OR placebo* OR (random* AND (allocat* OR assign*)) OR (blind* AND (single OR double OR treble OR triple)))
- WOS.IC: 1993 to 2025<br>- WOS.CCR: 1985 to 2025<br>- WOS.SCI: 1900 to 2025<br>- WOS.AHCI: 1975 to 2025<br>- WOS.ESCI: 2020 to 2025<br>- WOS.ISTP: 1990 to 2025<br>- WOS.SSCI: 1956 to 2025<br>- WOS.ISSHP: 1990 to 2025	TS=(animal or animals or pisces or fish or fishes or catfish or catfishes or sheatfish or silurus or arius or heteropneustes or clarias or gariepinus or fathead minnow or fathead minnows or pimephales or promelas or cichlidae or trout or trouts or char or chars or salvelinus or salmo or oncorhynchus or guppy or guppies or millionfish or poecilia or goldfish or goldfishes or carassius or auratus or mullet or mullets or mugil or curema or shark or sharks or cod or cods or gadus or morhua or carp or carps or cyprinus or carpio or killifish or eel or eels or anguilla or zander or sander or lucioperca or stizostedion or turbot or turbots or psetta or flatfish or flatfishes or plaice or pleuronectes or platessa or tilapia or tilapias or oreochromis or sarotherodon or common sole or dover sole or solea or zebrafish or zebrafishes or danio or rerio or seabass or dicentrarchus or labrax or morone or lamprey or lampreys or petromyzon or pumpkinseed or pumpkinseeds or lepomis or gibbosus or herring or clupea or harengus or amphibia or amphibian or amphibians or anura or salientia or frog or frogs or rana or toad or toads or bufo or xenopus or laevis or bombina or epidalea or calamita or salamander or salamanders or newt or newts or triturus or reptilia or reptile or reptiles or bearded dragon or pogona or vitticeps or iguana or iguanas or lizard or lizards or anguis fragilis or turtle or turtles or snakes or snake or aves or bird or birds or quail or quails or coturnix or bobwhite or colinus or virginianus or poultry or poultries or fowl or fowls or chicken or chickens or gallus or zebra finch or taeniopygia or guttata or canary or canaries or serinus or canaria or parakeet or parakeets or grasskeet or parrot or parrots or psittacine or psittacines or shelduck or tadorna or goose or geese or branta or leucopsis or woodlark or lullula or flycatcher or ficedula or hypoleuca or dove or doves or geopelia or cuneata or duck or ducks or greylag or graylag or anser or harrier or circus pygargus or red knot or great knot or calidris or canutus or

	godwit or limosa or lapponica or meleagris or gallopavo or jackdaw or corvus or monedula or ruff or philomachus or pugnax or lapwing or peewit or plover or vanellus or swan or cygnus or columbianus or bewickii or gull or chroicocephalus or ridibundus or albifrons or great tit or parus or aythya or fuligula or streptopelia or risoria or spoonbill or platalea or leucorodia or blackbird or turdus or merula or blue tit or cyanistes or pigeon or pigeons or columba or pintail or anas or starling or sturnus or owl or athene noctua or pochard or ferina or cockatiel or nymphicus or hollandicus or skylark or alauda or tern or sterna or teal or crecca or oystercatcher or haematopus or ostralegus or shrew or shrews or sorex or araneus or crocidura or russula or european mole or talpa or chiroptera or bat or bats or eptesicus or serotinus or myotis or dasycneme or daubentonii or pipistrelle or pipistrellus or cat or cats or felis or catus or feline or dog or dogs or canis or canine or canines or otter or otters or lutra or badger or badgers or meles or fitchew or fitch or foumart or foulmart or ferrets or ferret or polecat or polecats or mustela or putorius or weasel or weasels or fox or foxes or vulpes or common seal or phoca or vitulina or grey seal or halichoerus or horse or horses or equus or equine or equidae or donkey or donkeys or mule or mules or pig or pigs or swine or swines or hog or hogs or boar or boars or porcine or piglet or piglets or sus or scrofa or llama or llamas or lama or glama or deer or deers or cervus or elaphus or cow or cows or bos taurus or bos indicus or bovine or bull or bulls or cattle or bison or bisons or sheep or sheeps or ovis aries or ovine or lamb or lambs or mouflon or mouflons or goat or goats or capra or caprine or chamois or rupicapra or leporidae or lagomorpha or lagomorph or rabbit or rabbits or oryctolagus or cuniculus or laprine or hares or lepus or rodentia or rodent or rodents or murinae or mouse or mice or mus or musculus or murine or woodmouse or apodemus or rat or rats or rattus or norvegicus or guinea pig or guinea pigs or cavia or porcellus or hamster or hamsters or mesocricetus or cricetulus or cricetus or gerbil or gerbils or jird or jirds or meriones or unguiculatus or jerboa or jerboas or jaculus or chinchilla or chinchillas or beaver or beavers or castor fiber or castor canadensis or sciuridae or squirrel or squirrels or sciurus or chipmunk or chipmunks or marmot or marmots or marmota or suslik or susliks or spermophilus or cynomys or cottonrat or cottonrats or sigmodon or vole or voles or microtus or myodes or glareolus or primate or primates or prosimian or prosimians or lemur or lemurs or lemuridae or loris or bush baby or bush babies or bushbaby or bushbabies or galago or galagos or anthropoidea or anthropoids or simian or simians or monkey or monkeys or marmoset or marmosets or callithrix or cebuella or tamarin or tamarins or saguinus or leontopithecus or squirrel monkey or squirrel monkeys or saimiri or night monkey or night monkeys or owl monkey or owl monkeys or douroucoulis or aotus or spider monkey or spider monkeys or ateles or baboon or baboons or papio or rhesus monkey or macaque or macaca or mulatta or cynomolgus or fascicularis or green monkey or green monkeys or chlorocebus or vervet or vervets or pygerythrus or hominoidea or ape or apes or hylobatidae or gibbon or gibbons or siamang or siamangs or nomascus or symphalangus or hominidae or orangutan or orangutans or pongo or chimpanzee or chimpanzees or pan troglodytes or

	bonobo or bonobos or pan paniscus or gorilla or gorillas or troglodytes)
- WOS.IC: 1993 to 2025<br>- WOS.CCR: 1985 to 2025<br>- WOS.SCI: 1900 to 2025<br>- WOS.AHCI: 1975 to 2025<br>- WOS.ESCI: 2020 to 2025<br>- WOS.ISTP: 1990 to 2025<br>- WOS.SSCI: 1956 to 2025<br>- WOS.ISSHP: 1990 to 2025	#3 NOT #4
- WOS.IC: 1993 to 2025<br>- WOS.CCR: 1985 to 2025<br>- WOS.SCI: 1900 to 2025<br>- WOS.AHCI: 1975 to 2025<br>- WOS.ESCI: 2020 to 2025<br>- WOS.ISTP: 1990 to 2025<br>- WOS.SSCI: 1956 to 2025<br>- WOS.ISSHP: 1990 to 2025	TS=("as needed" or "as-needed" or prn or "pro re nata" or "on demand" or "on-demand" or "as required" or rescue)
- WOS.IC: 1993 to 2025<br>- WOS.CCR: 1985 to 2025<br>- WOS.SCI: 1900 to 2025<br>- WOS.AHCI: 1975 to 2025<br>- WOS.ESCI: 2020 to 2025<br>- WOS.ISTP: 1990 to 2025<br>- WOS.SSCI: 1956 to 2025<br>- WOS.ISSHP: 1990 to 2025	TS=(maintenance or continuous or continued)

- WOS.IC: 1993 to 2025<br>- WOS.CCR: 1985 to 2025<br>- WOS.SCI: 1900 to 2025<br>- WOS.AHCI: 1975 to 2025<br>- WOS.ESCI: 2020 to 2025<br>- WOS.ISTP: 1990 to 2025<br>- WOS.SSCI: 1956 to 2025<br>- WOS.ISSHP: 1990 to 2025	#1 AND #2 AND #5 AND (#6 OR #7)

Central

#1 (azelastine* OR azelastine-fluticasone OR azeflu OR mp-azeflu OR (azelastine AND fluticasone) OR dymista OR azecort OR olopatadin* OR olopatadine-mometasone OR (olopatadine AND mometasone) OR gsp301 OR ryaltris OR levocabastin* OR lexicomp OR ciclesonid* OR alvesco OR fluticason* OR gw685698x OR mometason* OR beclometason* OR budesonid* OR triamcinolon* OR antazoline* or methapyrilene* OR pyrilamine* OR tripelennamine* OR clemastine* OR dimenhydrinate* OR diphenhydramine* OR doxylamine* OR brompheniramine* OR chlorpheniramine* OR dimethindene* OR pheniramine* OR triprolidine* OR promethazine* OR cetrizine* OR meclizine* OR hydroxyzine* OR astemizole* OR cyproheptadine* OR loratadine* OR terfenadine* OR acrivastine* OR fexofenadine* OR dexbrompheniramine* OR carbinoxamine* OR bilastine* OR cetirizine* OR desloratadine* OR dexchlorpheniramine* OR dimetindene* OR diphenhydramine* OR ebastine* OR epinastine* OR levocabastine* OR rupatadine* OR mizolastine* OR mequitazine* OR alimemazine* OR trimeprazine* OR oxatomide* OR oxomemazine* OR quifenadine*):ti,ab,kw 24766

#2 MeSH descriptor: [Olopatadine Hydrochloride] explode all trees 137

#3 MeSH descriptor: [Fluticasone] explode all trees 1989

#4 MeSH descriptor: [Mometasone Furoate] explode all trees 529

#5 MeSH descriptor: [Beclomethasone] explode all trees 1222

#6 MeSH descriptor: [Budesonide] explode all trees 2229

#7 MeSH descriptor: [Triamcinolone] explode all trees 1829

#8 MeSH descriptor: [Astemizole] explode all trees 120

#9 MeSH descriptor: [Cetirizine] explode all trees 621

#10 MeSH descriptor: [Chlorpheniramine] explode all trees 296

#11 MeSH descriptor: [Cyproheptadine] explode all trees 738

#12 MeSH descriptor: [Clemastine] explode all trees 116
#13 MeSH descriptor: [Hydroxyzine] explode all trees 877
#14 MeSH descriptor: [Loratadine] explode all trees 534
#15 MeSH descriptor: [Ketotifen] explode all trees 260
#16 MeSH descriptor: [Promethazine] explode all trees 462
#17 MeSH descriptor: [Terfenadine] explode all trees 590
#18 MeSH descriptor: [Antazoline] explode all trees 21
#19 MeSH descriptor: [Methapyrilene] explode all trees 3
#20 MeSH descriptor: [Pyrilamine] explode all trees 24
#21 MeSH descriptor: [Tripelennamine] explode all trees 7
#22 MeSH descriptor: [Dimenhydrinate] explode all trees 124
#23 MeSH descriptor: [Diphenhydramine] explode all trees684
#24 MeSH descriptor: [Doxylamine] explode all trees 50
#25 MeSH descriptor: [Brompheniramine] explode all trees 134
#26 MeSH descriptor: [Dimethindene] explode all trees 54
#27 MeSH descriptor: [Pheniramine] explode all trees 452
#28 MeSH descriptor: [Triprolidine] explode all trees 81
#29 MeSH descriptor: [Meclizine] explode all trees 31
#30 MeSH descriptor: [Trimeprazine] explode all trees 43
#31 MeSH descriptor: [Histamine H1 Antagonists] explode all trees 1943
#32 #1 OR #2 OR #3 OR #4 OR #5 OR #6 OR #7 OR #8 OR #9 OR #10 OR #11 OR #12 OR #13 OR #14 OR #15 OR #16 OR #17 OR #18 OR #19 OR #20 OR #21 OR #22 OR #23 OR #24 OR #25 OR #26 OR #27 OR #28 OR #29 OR #30 OR #31 26103
#33 (rhinit* OR rhinoconjunctivit*):ti,ab,kw 12357
#34 (hayfever OR "hay fever" OR pollenosis OR pollinosis OR "SAR"):ti,ab,kw 2087
#35 MeSH descriptor: [Rhinitis] explode all trees 5056
#36 MeSH descriptor: [Rhinitis, Allergic] explode all trees 3796
#37 #33 OR #34 OR #35 OR #36 13243
#38 #32 AND #37 3977
#39 ("as needed" OR as-needed OR prn OR "pro re nata" OR "on demand" OR "on-demand" OR "as required" OR rescue):ti,ab,kw 42782
#40 (maintenance OR continuous OR continued):ti,ab,kw 170144
#41 #38 AND (#39 OR #40) 338
